# The availability of emergency obstetric care in the context of the JSY cash transfer programme in Madhya Pradesh, India

**DOI:** 10.1186/s12884-016-0896-x

**Published:** 2016-05-18

**Authors:** Yogesh Sabde, Vishal Diwan, Bharat Randive, Sarika Chaturvedi, Kristi Sidney, Mariano Salazar, Ayesha De Costa

**Affiliations:** Department of Community Medicine, R.D. Gardi Medical College, Ujjain, India; Department of Public Health Sciences, Karolinska Institutet, Stockholm, Sweden; Department of Public Health and Environment, R.D. Gardi Medical College, Ujjain, India; International Centre for Health Research, R.D. Gardi Medical College, Ujjain, India; Epidemiology and Global Health, Department of Public Health and Clinical Medicine, Umeå University, Umeå, Sweden

## Abstract

**Background:**

Since 2005, India has implemented a national cash transfer programme, the Janani Suraksha Yojana (JSY), which provides women a cash transfer upon giving birth in an existing public facility. This has resulted in a steep rise in facility births across the country. The early years of the programme saw efforts being made to strengthen the ability of facilities to provide obstetric care. Given that the JSY has been able to draw millions of women into facilities to give birth (there have been more than 50 million beneficiaries thus far), it is important to study the ability of these facilities to provide emergency obstetric care (EmOC), as the functionality of these facilities is critical to improved maternal and neonatal outcomes. We studied the availability and level of provision of EmOC signal functions in public facilities implementing the JSY programme in three districts of Madhya Pradesh (MP) state, central India. These are measured against the World Health Report (WHR) 2005benchmarks. As a comparison, we also study the functionality and contribution of private sector facilities to the provision of EmOC in these districts.

**Methods:**

A cross-sectional survey of all healthcare facilities offering intrapartum care was conducted between February 2012 and April 2013. The EmOC signal functions performed in each facility were recorded, as were human resource data and birth numbers for each facility.

**Results:**

A total of 152 facilities were surveyed of which 118 were JSY programme facilities. Eighty-six percent of childbirths occurred at programme facilities, two thirds of which occurred at facilities that did not meet standards for the provision basic emergency obstetric care. Of the 29 facilities that could perform caesareans, none could perform all the basic EmOC functions. Programme facilities provided few EmOC signal functions apart from parenteral antibiotic or oxytocic administration. Complicated EmOC provision was found predominantly in non-programme (private) facilities; only one of six facilities able to provide such care was in the public sector and therefore in the JSY programme. Only 13 % of all qualified obstetricians practiced at programme facilities.

**Conclusions:**

Given the high proportion of births in public facilities in the state, the JSY programme has an opportunity to contribute to the reduction in maternal and perinatal mortality However, for the programme to have a greater impact on outcomes; EmOC provision must be significantly improved.. While private, non-programme facilities have better human resources and perform caesareans, most women in the state give birth under the JSY programme in the public sector. A demand-side programme such as the JSY will only be effective alongside an adequate supply side (i.e., a facility able to provide EmOC).

## Background

Reports have shown that the global maternal mortality ratio (MMR) has been falling since 1990; however, this reduction has shown variations across and within regions. Half of all maternal deaths worldwide were concentrated in six countries, including India [1]. Although 18 % of global maternal deaths take place in India, the country has seen a steady decline in maternal mortality [2]. In 2013, the MMR in India was 190 per 100,000 live births [3].

The prioritisation of the intrapartum period is central to any strategy that aims to reduce maternal mortality [4]. A health centre-based intrapartum care strategy has been recommended as an effective means of reducing high maternal mortality, as most maternal deaths occur during labour, birth or the first 24 h post-partum due to complications that cannot always be predicted or prevented [4]. Facility births are assumed to facilitate skilled birth attendance and access to life-saving emergency obstetric care (EmOC) with which complications can be appropriately managed. In 2005, India promoted an institutional birth strategy under its National Rural Health Mission [5]. In much of the country, this strategy was implemented through the Janani Suraksha Yojana (JSY) programme, a conditional cash transfer targeted to women giving birth in public facilities. The JSY programme has had over 50 million beneficiaries [6] since its inception in 2005 [7]. It has been successful in raising institutional birth proportions across the country, from 38 % in 2005 to 74 % in 2013 [8]. However, studies have not been able to detect any significant reduction in maternal mortality that is attributable to the programme [9, 10].

While much has been written about the JSY programme’s success in increasing facility births, little has been reported on the functionality of the programme facilities themselves. The aim of the JSY has not been to increase the number of facilities but to promote births within existing public facilities. However, efforts made under the National Rural Health Mission in the early years of the JSY have aimed to strengthen the provision of care in facilities by providing skilled birth attendance training to staff, increasing the availability of equipment and supplies and recruiting more staff. If the JSY is to meet its ultimate goal of reducing maternal mortality, the ability of the facilities to perform key signal functions that constitute EmOC [11] is critical. Given that the JSY has been able to incentivise millions of women to give birth in public facilities, it is important to study the ability of these facilities to provide EmOC to these women. In this paper, we study the functionality of JSY programme facilities in three districts of Madhya Pradesh (MP) state, central India. MP has had the highest uptake of the JSY programme in the country. As a comparison, we also look at the smaller private obstetric sector (non-programme) found in these districts. Specifically, this paper reports on (i) the status of EmOC provision and the human resources available to perform key EmOC functions in public facilities (all of which implement the JSY) and in private sector facilities and (ii) the availability and geographic distribution of public and private EmOC facilities in these districts.

## Methods

### Study setting

**Fig. 1:**
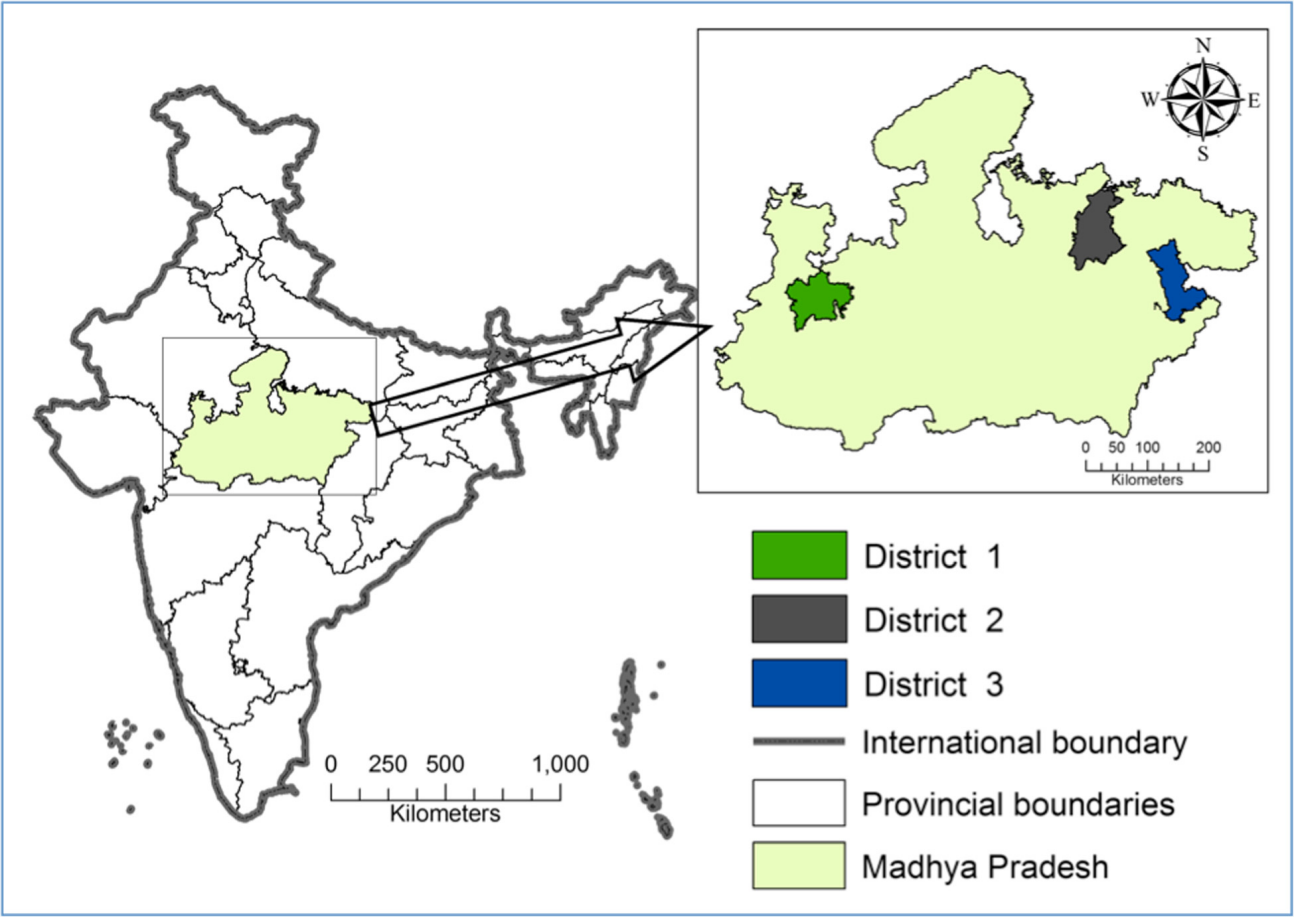
Study setting. The map depicted in Fig. 1 has been developed by the study team

The study was conducted in three districts of MP state, central India (Fig. 1). MP has a population of 72 million. It has a relatively high birth rate of 24.8/1000 [12]. In 2012, the MMR was 277 per 100,000 live births, but it varied from 202 to 415 across districts [12]. The state has had one of the highest utilisation rates of the JSY programme nationwide [9]. In 2009, 86 % of women in MP were aware of the JSY programme, 72 % had had institutional births and 67.8 % had given birth at public health care facilities [13].

In India, districts are administrative divisions into which a state is divided. Each of MP’s 51districts has its own health administration to manage public health facilities within the district. The three districts in this study (Fig. 1) were selected to reflect (a) different geographic regions of MP, (b) different sociocultural zones of MP, (c) different population subgroups, with districts 1,2 and 3 having a predominance of urban, rural and tribal populations, respectively, and (d) different socio economic levels, with district 1 being relatively more affluent than districts 2 and 3 (Table 1). The total population of the study area was 4.07 million people.

**Table 1:** Profiles of study districts

Characteristics	Madhya Pradesh	District 1	District 2	District 3
Population (in millions)^a^	72.0	1.9	1.02	1.07
Proportion rural population^a^	68.0	58.0	88.0	71.0
Birth rate per 1000^b^	24.8	24.0	31.5	24.2
Institutional delivery %^b^	76.0	81.0	72.0	58.0
Human Development Index 2007^c^	0.4	0.6	0.5	0.6
MMR^a^	277.0	206	386.0	415.0

^a^Government of India (2011) Provisional Population Totals: Madhya Pradesh Census

^b^Government of India (2012) Annual Health Survey Bulletin 2011–2012: Madhya Pradesh. New Delhi

^c^Maternal mortality ratio. Government of Madhya Pradesh, Madhya Pradesh Human Development Report (2007), Government of Madhya Pradesh: Bhopal

### Data collection

Public and private health facilities that attended to at least one birth a month were identified from a list provided by district-level authorities. These facilities were visited by trained researchers between February 2012 and April 2013. Respondents from these facilities were asked to provide information on any other facilities they knew in the area that were not in the original list. This snowball technique was used to complement the original list of facilities that researchers visited.

Advance permission for facility visits was sought from the state and district health authorities. Permission was also sought from the administrative head of the facility concerned. The survey included the following elements:

(i) A short interview was conducted with the administrator of the facility. This interview elicited information about the facility’s basic characteristics, including the number of beds, and information on the type (by qualification) and number of human resources available for skilled birth attendance at each facility. In small facilities (primary- or secondary-level facilities), all available beds were counted. In larger district level hospitals that have a large number of specialty departments, only obstetric ward beds were counted.
(ii) A short interview with the staff member in-charge of the obstetric ward was conducted to obtain information about the performance of the seven basic and two comprehensive EmOC functions at the facility, specifically during the last three months. In smaller facilities, the doctor in charge of the facility provided this information. In case of non-performance of any of these functions, the reasons for non-performance at the particular facility were elicited.
(iii) The number of births in each facility in the last three months prior to the date of the survey were obtained from facility registers at each facility.
(iv) Further, to assess EmOC service coverage, each facility surveyed was geo-referenced using global positioning systems (GPS). The geo-referenced data of the study districts were entered in ArcMap version 10. For geo-referencing, Survey of India topographic maps (1:50,000 scale) were used [14]. Readings from handheld GPS taken at four major crossroads in each district were used to cross-check geo-referencing in the topographic maps. The geo-referenced data included 1) the digital boundary maps of the study districts, which were retrieved from the Survey of India office, and 2) locations of all facilities included in this study.

Facilities were generally visited once; however, due to logistic reasons, in some cases two visits needed to be made to obtain the above information. When a second visit was needed, it was made most often within a week of the first visit.

#### Assessing EmOC signal functions and classifying facilities

We used the United Nations (UN) handbook on monitoring EmOC to assess the performance of listed EmOC signal functions during the three month period prior to the date on which the facility was visited [11]. In the case of non-performance of EmOC services in any facility surveyed, the reasons for non-performance were classified, as indicated in the UN handbook, as training issues, lack of supplies or equipment, drug issues, management issues, policy issues, no indication and others [11]. The UN benchmark on the recommended number of comprehensive emergency obstetric care (CEmOC) facilities in a population has been criticized because it uses population as a denominator without taking into consideration birth rates. Thus, the benchmark does not specify the number of births that can be managed at each facility or the number of staff required at each facility to manage the number of deliveries [15]. Thus, for these indicators, we used the World Health Report (WHR) 2005 benchmarks [16], which take into consideration the number of births occurring in a population. The WHR 2005 explicitly asserts that ‘all mothers and new-borns, not just those considered to be at particular risk of developing complications, need skilled maternal and neonatal care’ [16], and the report consequently sets its benchmarks in order to meet this need. Our reference population included the populations of the three study districts, as listed in the Census of India. To estimate the number of births in our three districts, we applied the crude birth rates available from a national survey by the Registrar General of India [12]. We also assumed that all women should have access to birth in an EmOC facility. This is in line with the ‘skilled attendance for all’ approach, which argues that most complications are unpredictable and many need quick attention [4], and with the logic of the JSY programme, which aims to promote facility births to decrease maternal mortality [16].

### Classifying facilities

Program and non-program facilities providing *all* seven basic EmOC signal functions (administration of parenteral antibiotics, uterotonic drugs and parenteral anticonvulsants for eclampsia, manual removal of the placenta, removal of retained products of conception, assisted vaginal delivery and neonatal resuscitation) were classified as basic emergency obstetric care (BEmOC) facilities. Those that did not perform all seven basic functions were classified as ‘less-than-BemOC’ facilities.

Facilities that performed all basic signal functions *and* provided caesarean sections and blood transfusions were classified as ‘comprehensive emergency obstetric care’ facilities (CEmOC).

In addition to the above, we found a large number of facilities that provided caesarean section services but failed to provide the other eight EmOC signal functions. These were classified as ‘less-than-CEmOC’ facilities.

### Analysis

A database was created using REDCap (Research Electronic Data Capture, a web application for building and managing databases for research studies) [17] and exported to Stata version 12 (StataCorp, College Station, Texas) for analysis. Descriptive statistics (frequencies, percentages, medians and inter quartile ranges (IQR), maps and spider diagrams) have been used to present the data. Medians and IQR were used because the continuous variables were not normally distributed.

### Ethical approval

Ethical approval to conduct the study was obtained from the Institutional Ethical Review Board at R. D. Gardi Medical College, Ujjain, MP, India.

## Results

### Facility characteristics

We surveyed a total of 929 facilities (386,188 and 355 in districts 1,2 and 3, respectively). Of these, 157 (118 programme and 39 non-programme) attended to at least one childbirth per month and were included in the study (37,27 and 35 in districts 1,2 and 3 respectively). Five of the facilities (all non-programme) that met these inclusion criteria refused to participate and were excluded from the study. In total, we obtained data for 118 programme facilities and 34 non-programme facilities.

None of the facilities (programme or non-programme) met the criteria to be classified as an exclusively BEmOC facility. Of the 118 programme facilities, 113 were classified as less-than-BEmOC, four as less-than-CEmOC and one as CEmOC (Table 2). Of the 34 non-programme facilities, four were classified as less-than-BEmOC, 25 as less-than-CEmOC and five as CEmOC (Table 3).

**Table 2:** Number of facilities and the deliveries conducted in the studied programme facilities during last three months according to their EmOC status

	Programme facilities					
	Number of facilities	Deliveries conducted	Total bed strength	Skilled birth attendants		
				Obstetrician/Gynaecologist^b^	Non specialist Doctors	Nurses/ANM
Facility level	n	n	n	n	n	n
CEmOC	1	642	30	2	3	7
Less-than- CEmOC	4	4502	270	2	8	34
Less-than- BEmOC	113^a^	10157	668	5	105	338
Total	118	15301	968	9	116	379

*n* = 118

^a^Include 40 sub-centres, 49 primary centres, 17 community health centres and 7 Sub district hospitals. No health facility was classified as BEmOC. ^b^This variable was collected in facilities that performed more than 10 deliveries in a month (*n* = 74)

**Table 3:** Number of facilities and the deliveries conducted in the studied non-programme facilities during last three months according to their EmOC status

	Non-programme facilities					
	Number of facilities	Deliveries conducted	Total bed strength	Skilled birth attendants		
				Obstetrician/Gynaecologist^a^	Doctor	Nurse/ANM
Facility level	n	n	n	n	n	N
CEmOC	5	1002	148	38	26	24
Less-than-CEmOC	25	1380	298	20	50	99
Less-than- BEmOC	4	32	48	-	7	11
Total	34	2414	494	58	83	134

*n* = 34

^a^This variable was collected in facilities that performed more than 10 deliveries in a month (*n* = 22)

### Distribution of births by facility type (programme/non-programme) and level of EmOC provision

Most births during the study period (February 2012 and April 2013) occurred at programme facilities (86 %, *n* = 15,301). Of those who gave birth in programme facilities, six out of ten births were in less-than-BEmOC facilities (Table 2). Among programme facilities, the median number of deliveries over three months in less-than-BEmOC facilities was 41 (IQR 11 – 115), while the median was higher in facilities providing caesarean section (CS) services, at 658 (IQR 642 – 1188) births.

Nine percent (*n* = 1644) of all births occurred in CEmOC facilities (Table 4). Of these births, 40 % occurred in the lone programme CEmOC facility, while the remaining 60 % of births were distributed among the five non-programme CEmOC facilities. A third of all births (*n* = 5,846) occurred in less-than-CEmOC facilities (Table 4), and three times as many births occurred in the four programme-affiliated less-than-CEmOC facilities than in the 25 non-programme ‘less-than-CEmOC’ facilities.

**Table 4:** Proportion of all facility births occurring at different levels within and outside the programme

Facility level	Programme (*n* = 15301)	Private (*n* = 2414)	Total (*n* = 17715)
CEmOC	4 %	42 %	9 %
Less-than-CEmOC	30 %	57 %	33 %
Less-than-BEmOC	66 %	1 %	58 %
Total	100 %	100 %	100 %

In the non-programme facilities, 99 % of births occurred in either CEmOC or less-than-CEmOC facilities (Table 4). Nearly all births in less-than-BEmOC facilities occurred within the programme.

### Bed strength

Two thirds of the nearly 1,500 obstetric beds in the district were found in programme facilities. In programme facilities, the median number of beds in a less-than-BEmOC facility was 2 (IQR 1 – 6), while facilities with CS services had 34 (IQR 30–48) beds (data not shown). Non-programme facilities with CS services had a median bed strength of 10 (IQR6 – 20) beds.

### Human resources

Of the 67 obstetricians working in the three districts, only nine were employed in programme facilities. In programme facilities, these nine obstetricians were distributed across different levels of EmOC provision (Table 2), while in the non-programme facilities58 obstetricians were present in facilities at the CEmOC and less-than-CEmOC levels (Table 3). Non-specialist doctors were present in both programme and non-programme facilities, as seen in Tables 2 and 3. Among the programme facilities, non-specialist doctors were concentrated at less-than-BEmOC facilities, where they were also responsible for administrative and clinical oversight of the facility (Table 2). Nurses and auxiliary nurse-midwives (ANM) most often worked in the labour room with routine intrapartum care. There was a concentration of nurses and ANMs in primary health care facilities in the programme, as they staffed peripheral sub centres attached to primary health centers and worked at primary/secondary care facilities as well (less-than-BEmOC) (Table 2).

#### The availability of EmOC

In all districts, the overall availability of CEmOC was lower than that indicated by WHR benchmarks (Table 5). The gap in availability varied widely, with one district having no CEmOC facilities at all, while in another the CEmOC facilities were outside the public sector and hence outside the programme. Besides these CemOC facilities, there were no facilities that provided all 7 BEmOC functions in any of the districts. The less-than-BEmOC facilities provided less than four basic signal functions. The less-than-CEmOC facilities were also technically less-than-BEmOC level, as they did not provide all seven basic functions despite having the ability to perform CS.

**Table 5:** Availability of EmOC against WHR 2005 benchmarks

Indicator source	WHR 2005 benchmark	District 1	District 2	District 3
Births per year^c^		47760	32130	25894
Required CEmOC facilities	At least 1 per 3600 births	13–14	8–9	7–8
Total CEmOC facilities		5	1	0
Programme CEmOC facilities		0	1	0
Staffing				
Required midwives as per standard	20 midwives per 3600 births	265	178	144
Available midwives (equivalent)		207	118	188
Required doctors as per standard	3 doctors part-time per 3600 births	41	27	22
Total available doctors		183	35	88
Obstetricians/Gynaecologists		54	2	11
Doctors		99	31	69
Proportion of births needing CEmOC	17–18 %			
Proportion of births in CEmOC facilities				
Total		49 %	14 %	0 %
Programme		0 %	14 %	---
Non-programme		49 %	0 %	---
Births per midwife/equivalent per year^a^	175			
At CEmOC level		165	368	-
At less-than-CEmOC level		169	-	201
At less-than-BEmOC level		117	147	96
Complicated births per doctor (specialist)	200	^b^	^b^	^b^

Programme and non-programme facilities included

^a^Assuming all births delivered by this cadre. ^b^Not determined. ^c^Office of The Registrar and Census Commissioner, Annual Health Survey Bulletin 2011–12. Madhya Pradesh 2012, Government of India, New Delhi, India

There was a shortage of nurse-midwives in districts 1 and 2; their availability varied from 78 % (district 1) to 66 % (district 2) of the required numbers as per the WHR benchmarks (Table 5). However, the distribution of nurse-midwives varied by the level of care, as did the number of births per nurse-midwife. On average, the number of births per nurse-midwife per year fell short of WHR levels at the less-than-BEmOC facilitiesin all districts and at the CEmOC and less-than--CEmOC facilities in district 1. The number of births per nurse-midwife per year exceeded benchmarks at the CEmOC facilities in district 2 and at the less-than-CEmOC facilities in district 2 and district 3 (Table 5). Non-specialist physicians met the WHR benchmarks; however, they were also found to perform administrative tasks that diminished the time spent with patients (Table 5).

#### Performance of EmOC signal functions

**Fig. 2:**
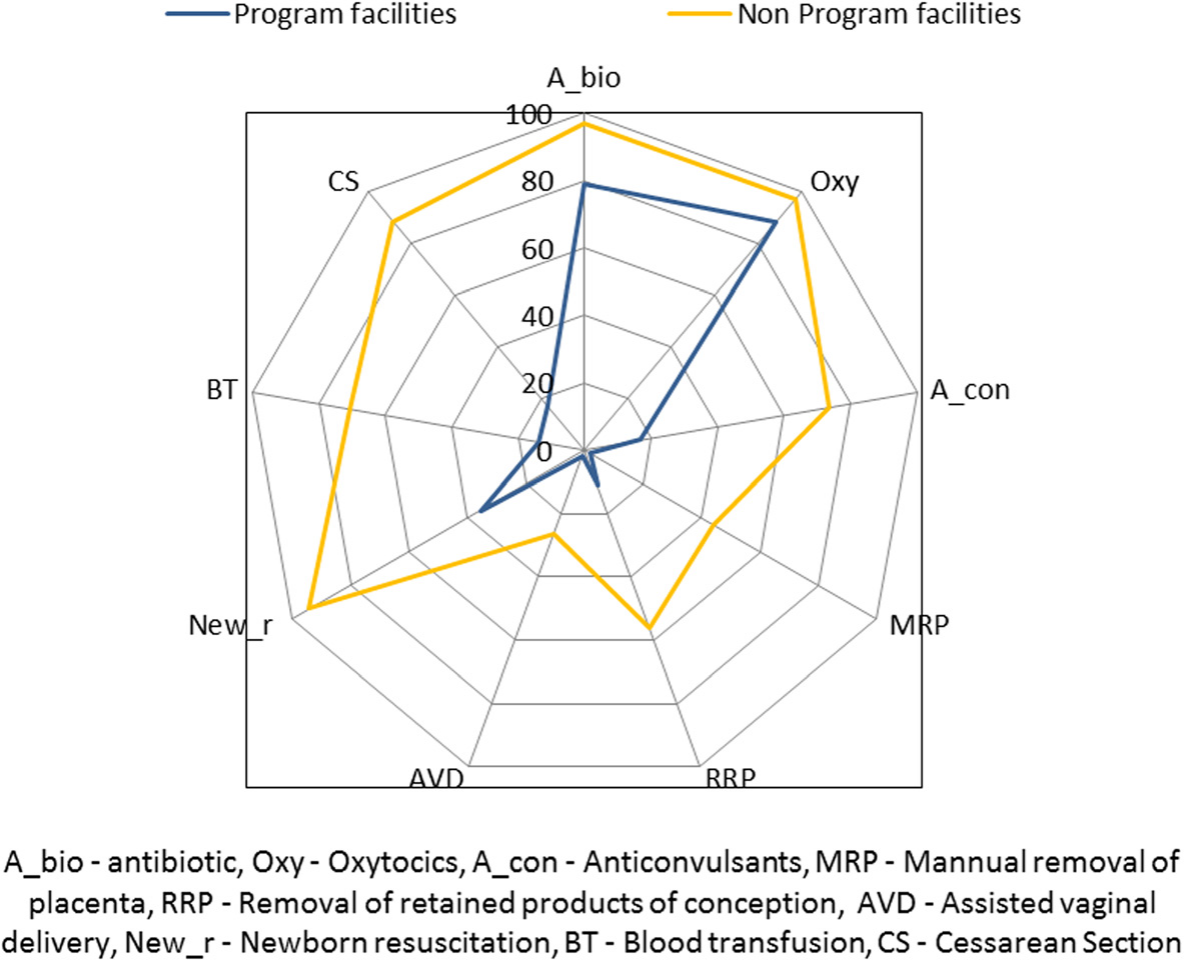
Distribution of EmOC performance of programme and non-programme facilities in the study districts

Eighty percent of programme facilities were able to administer parenteral antibiotics and oxytocics. However, the other basic EmOC signal functions were performed by less than 20 % of programme facilities assessed (Fig. 2). Assisted vaginal delivery and manual placental removal were performed at less than 2 % of programme facilities (Fig. 2). When comparing programme facilities to non-programme facilities, a higher proportion of non-programme facilities could perform most signal functions (Fig. 2).

Lack of supplies and insufficient training were the two most common reasons cited for not performing signal functions at programme facilities. Lack of supplies was the most common reason cited for not administering parenteral antibiotics or oxytocics. Lack of training was more frequently mentioned as a reason for not performing manual placental removal, removal of retained products of conception or assisted vaginal delivery.

#### Location of facilities for childbirth

**Fig. 3:**
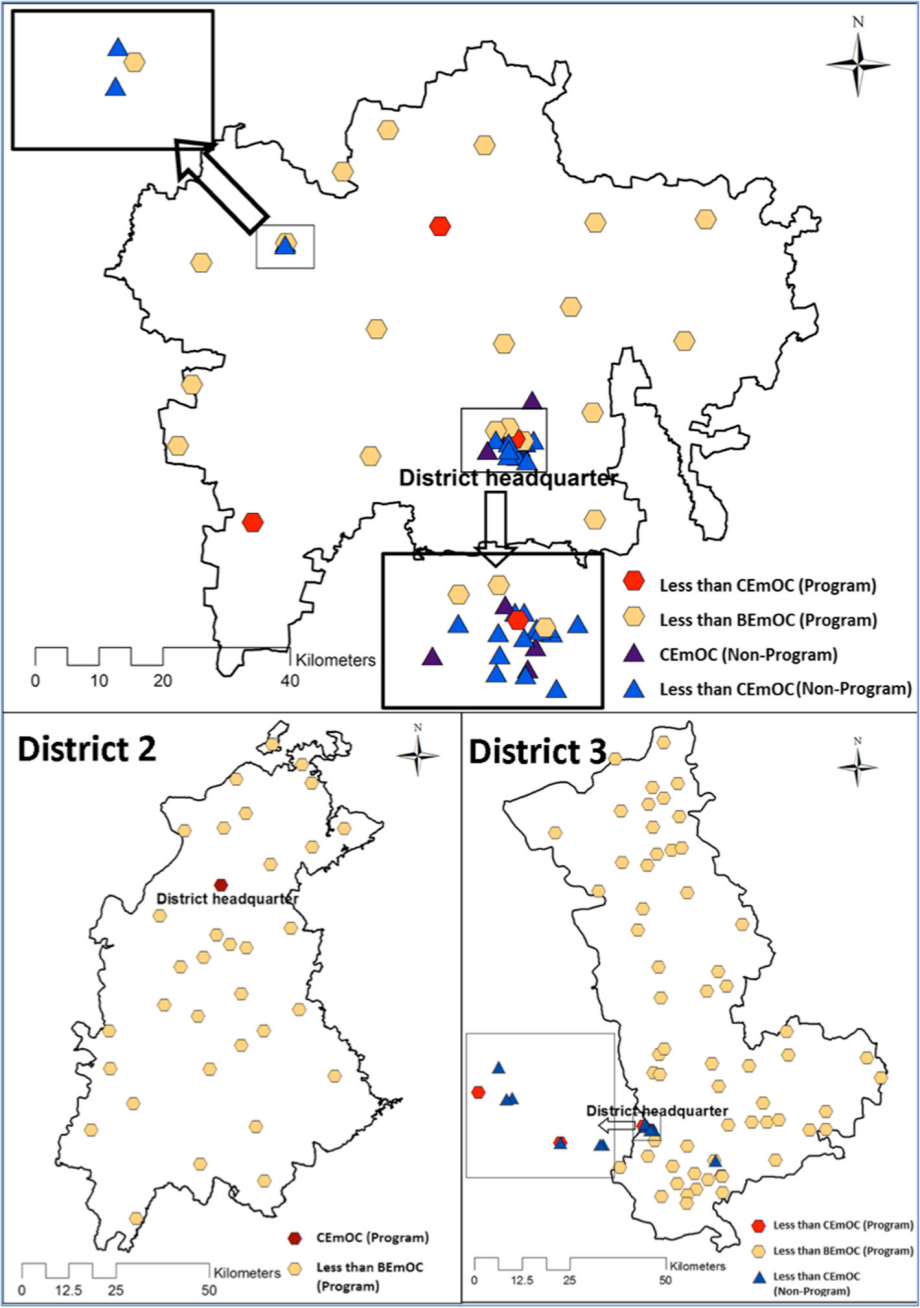
Performance of EmOC signal functions in programme and non-programme facilities in the study districts. The map depicted in Fig. 1 has been developed by the study team

Figure 3 shows that the less-than-BEmOC facilities are well-dispersed throughout the districts, such that all residents have access to one within a 20 km radius. However, as described above, the level of EmOC functioning of these facilities is poor in spite of their geographical accessibility. There are few less-than-CEmOC or CEmOC facilities in the programme (public) sector. The large majority of these facilities are in the non-programme (private) sector in the larger district headquarter towns.

## Discussion

In its Global Strategy for Women’s and Children’s Health, the World Health Organisation (WHO) highlighted that improving service delivery is a key strategy to improve maternal health and reduce maternal mortality [18]. Measuring output/process indicators (e.g., the availability of signal EmOC functions in health facilities and the geographical distribution of facilities offering EmOC) can be useful in illustrating a health system’s preparedness to reduce maternal mortality [13, 19]. It can be relatively more difficult to measure outcome and impact indicators [11, 20]. For example, the accurate measurement and reporting of maternal deaths are riddled with difficulties, particularly in low-income settings, and these measurements often have large confidence intervals. Our study reports on output indicators related to the provision of EmOC in both the programme (public) and non-programme (private) sectors in the context of the JSY cash transfer programme in three districts of Madhya Pradesh.

Our main findings show that although a majority of institutional births in three districts of Madhya Pradesh state occur in programme facilities, the delivery of EmOC services in these facilities is inadequate, as the facilities offer very few of the signal functions needed to prevent maternal mortality/morbidity. The number of births in each facility is sufficiently high to maintain skills, even in the less-than- BEmOC facilities. Non-programme (private sector) facilities performed a greater number of EmOC signal functions. CEmOC facilities were largely found in the private sector and were concentrated in the urban areas of the economically advantaged district (district 1), although even in these cases the overall availability of CEmOC was lower than required. Although just 14 % of institutional births occurred in non-programme facilities, the large majority of obstetricians worked in these non-programme facilities.

### The status of EmOC signal functions within the JSY programme

The programme facilities at all levels were primarily able to perform two signal functions: administering parenteral antibiotics and administering oxytocics. The availability of these functions could inaccurately be perceived as adequate to reduce maternal mortality as haemorrhage and sepsis are the two most common causes of maternal mortality in India [1, 21]. However, haemorrhage and sepsis during pregnancy are complex conditions that might need to be managed by calling into play any of the full array of signal functions (i.e., manual placental removal, removal of retained products of conception, assisted vaginal delivery or CEmOC functions such as blood transfusion or surgery) [22, 23].

With regard to basic EmOC functions, an issue of concern is that less than 20 % of the programme facilities in the study area were able to provide parenteral anticonvulsants. This is a key basic signal function that has been proven to save pregnant women’s lives. Preeclampsia and eclampsia are an important causes of maternal morbidity and mortality in MP [24–26] and elsewhere in India [1, 27–29]; 16 % of all maternal deaths in Madhya Pradesh are reported to result from these conditions [24]. The low capacity to perform this function implies that women with preeclampsia/eclampsia could be exposed to a phase III delay by not receiving appropriate health care after reaching a health facility [30].

The ability to perform any basic signal function that required some level of manual skill (i.e., manual placental removal, removal of retained products of conception or assisted vaginal delivery) was extremely low in all facilities surveyed in this study. Many of the facilities we classified as less-than-CEmOC had the ability to perform caesarean sections but could not perform these more basic functions. This is in line with a study that showed that in Nepal, Nicaragua and Honduras, a lower percentage of facilities classified as non-hospitals (lower level health centers) were able to perform these signal functions than those classified as hospitals [31], though all facilities in our study did have inpatient beds. However, although the performance of these functions by non-programme facilities was low, it was still much higher relative to programme facilities.

With regard to comprehensive emergency obstetric care functions, there was just one programme facility in the three districts that was qualified to be a fully functional CEmOC facility. Several large facilities (less-than-CEmOC) that could perform caesarean sections) were unable to perform the other basic signal functions, particularly the three basic functions referred to above. These facilities together take on a large proportion of total births. This may be in part because these are larger hospitals (in terms of bed and staff numbers), but it may also be because the low level of EmOC provided by less-than-BEmOC facilities leads to overcrowding. The proportion of complicated births carried out at these facilities needs to be studied. These facilities have a lack of human resources relative to the number of birth they attend to.

In general, the rates of use of parenteral antibiotics and oxytocin reported in this paper are similar to those reported by studies assessing the availability of emergency obstetric care functions in Zambia [32], Kenya [33] and six other African and Asian countries [34], regardless of health care facility ownership. However, in the programme health facilities assessed in this study, the performance rates of all other basic and comprehensive signal functions were below the proportions reported by these studies.

#### Reasons for not performing EmOC signal functions

The ability of a health system to reduce maternal mortality is strongly influenced by a range of factors, including availability, accessibility and quality of health services [35, 36]. The quality of health services is key to the effectiveness of demand-side interventions such as the JSY programme, as increased demand must be met with care that is of appropriate quality, including adequately trained human resources and carefully considered logistics [35]. This study shows that the reasons for non-performance of most EmOC signal functions at public JSY health facilities are often insufficient training and lack of supplies. This clearly indicates structural weaknesses in the health system that impact its ability to provide lifesaving EmOC [37] services during the intrapartum period when women are most at risk of death [4, 38]. The results presented in this paper are in line with evidence from studies in other low- and middle-income settings, which also found that insufficient training, lack of skilled staff and inadequate supplies impair the effective provision of both basic and comprehensive obstetric care [34, 39].

Key functions that could not be performed in most programme facilities included the three which required manual procedures, namely, manual placental removal, removal of retained products of conception and assisted vaginal delivery. While the performance of assisted vaginal delivery is decreasing in countries around the world [40], the lack of these skills on the whole indicate inadequate training of skilled birth attendants and the absence of supportive supervision for performance of these tasks. While our study reports a number of non-specialist doctors working at different levels in programme facilities, many of these individuals focus on administrative rather than clinical tasks and are not necessarily best-suited (or competent) to provide supportive clinical supervision. Our previous study found that only 20 % of the nurses working as skilled birth attendants in programme facilities in the three districts of Madhya Pradesh, India, were competent at providing first line of care to pregnant women with eclampsia or hemorrhage; and only one out of ten was considered competent to perform a correct initial assessment of women with these complications [41].

Forty percent of the referrals made by secondary facilities to tertiary programme health facilities in this setting were due to complications that should have been managed by the referring facility [42], and this may result from lack of skilled staff. In order to provide effective and appropriate EmOC in this setting, more emphasis needs to be placed on the strengthening of human resource competency. Efforts need to be focused on improved training (including continued education programmes) and increased monitoring (including periodical supervision, quality checks and accountability) for health workers [35, 37, 43].

The lack of supplies, reported in this paper as one of the major reasons for not performing obstetric emergency care signal functions, is an important barrier to the provision of effective health strategies in reducing maternal mortality. Kerber et al. [43] identified that management or financial issues of the supply chain might influence the availability of key drugs and equipment at health facilities. However, this study did not explore indepth the reasons behind the lack of supplies.

#### Number of births at programme facilities: Are the trade-offs between efficiency, quality and access relevant in this setting?

The WHR 2005 also briefly addressed the issue of geographical accessibility of facilities, proposing a larger number of smaller facilities for more dispersed populations and discussing the trade-offs between efficiency, quality and accessibility.

Balancing access with efficiency is particularly relevant in less dense populations; however, populations in our setting are rather dense, and programme facilities are sufficiently dispersed to provide geographical access, with no village further than 20 km from a facility. However, the functionality of facilities requires attention. The WHR 2005 benchmarks assume an average annual work load of 175 births per midwife [16], a figure based on the observed median of certain district hospitals in Sub-Saharan Africa [44]. However, different countries report widely varying numbers of births per midwife [45]. Data from neighbouring Sri Lanka [46], a country with a much lower maternal mortality, indicate that the number of deliveries per midwife there is very similar to the numbers reported in our study, which are close to the WHR 2005 benchmarks. This suggests that the number of births per nurse-midwife is not the problem in our setting. The literature mentions that non-performance of key EmOC functions often arises because births are too few for skills to be maintained, particularly skills required for manual procedures. However, this was not the case in our study. Given that the number of births per nurse-midwife is not low, midwifery skills, when available, are unlikely to be lost.

#### Pro-urban distribution of CEmOC facilities

Our study shows inequalities in both the availability and geographical distribution of CEmOC health facilities in the areas under study. CEmOC facilities in the study area were concentrated in the non-programme private sector and in richer urban areas. Taken together, the programme and non-programme provision of CEmOC did not meet the WHR 2005 benchmarks [16]. Our findings are in line with those of a recent study showing that the provision of CEmOC was highly privatized in eight districts of Karnataka state, India, in which nine out of the ten facilities providing CEmOC were privately owned [47]. India is known to have a highly privatized healthcare system, which users pay for out-of-pocket [48]. This is likely to contribute to the concentration of CEmOC in wealthier urban areas. The challenge of how to increase access to comprehensive obstetric care for rural and poor women remains. The state needs to focus its efforts on improving the quality of CEmOC provision in these areas, as the absence of any non-state providers makes women dependent on the public sector’s provision of life saving EmOC.

#### The private obstetric care sector: small but large

While the composition of the Indian private health sector and its relative size vary in different parts of the country, in Madhya Pradesh, it is much smaller than the state sector with regard to the provision of obstetric care. Even though the private sector provided care in just 14 % of all births recorded in our study, it employs the overwhelming majority of qualified obstetricians in the study area. The private sector is the larger sector in terms of the availability of specialist obstetric care, particularly access to CS. While most facilities in this sector can perform CS when necessary, only a small proportion can perform signal functions requiring manual skills.

Private obstetricians working in non-programme facilities practice largely in urban areas, where clients are able to pay out of pocket for their services. However, this creates financial barriers to access for many women, especially in a poor state such as Madhya Pradesh. Thus, even though the level of EmOC functioning is higher in the private sector than in the programme facilities, few women can access these higher-functioning services. In our study, only 14 % of births occurred in private facilities even though the private sector facilities contain 33 % of all beds. The JSY programme has made some attempts to partner with the private sector, but this has met with limited success.

### A comparison between the programme and non-programme facilities

Though programme facilities are widely available and geographically accessible, even to rural populations in MP, the functionality of these facilities requires significant improvement if they are to effectively reduce maternal mortality. Recruiting and retaining skilled specialist staff have been problematic for programme facilities despite a number of special incentives by the Department of Health in Madhya Pradesh. This compromises the ability of programme facilities to deliver competent EmOC.

Non-programme facilities have a comparatively better capacity to provide EmOC, given that they have specialist staff (obstetricians) and the ability to provide CS. In this respect, our setting differs from EmOC assessments conducted in other low-income settings, particularly in Sub-Saharan Africa. However, access to non-programme EmOC care is restricted by (a) financial barriers to access, as most payments are made out-of-pocket by users, and (b) geographic barriers to access, as these facilities tend to be concentrated in urban areas. Therefore, there is an increased importance and relevance for the JSY programme in the context of an area such as MP. However, implementing a large demand-side programme such as the JSY without adequately strengthening the supply side (e.g., ensuring the quality of care) will not have any significant impact on health outcomes, other than raising the numbers of hospital births because of the financial incentive. Further, providing financial incentives to vulnerable women to give birth in facilities that do not provide adequate standards of EmOC is also fraught with ethical concerns.

### Methodological considerations

Our conclusions are based on findings from three very different districts in Madhya Pradesh. Though the districts are heterogeneous, there are similarities with regard to the findings. However, other districts could yield somewhat different findings with regard to EmOC provision. The generalisability to other states in India is limited given the variation in the composition of the health system (public versus private) and other background characteristics such as socio-economic and infrastructural variables.

The choice to use the WHR 2005 benchmarks instead of the UN 2009 benchmarks was based on a critique of the two standards. We used the WHR 2005 benchmarks because they allowed a more complete picture of the provision of obstetric care that went beyond facility density. The WHR’s assumption that all births need access to EmOC is in line with the logic of the JSY programme.

Nurse-midwives in our setting have been equated to midwives in the WHR report, as this group is the major provider of skilled birth attendance and is trained to provide this care in the absence of a specialized midwifery profession in the country. It is possible that we have underestimated the number of births performed by each nurse-midwife in a facility, as a proportion of nurse-midwives are also involved in community work in the catchment area of public health facilities.

## Conclusions

In order to be able to reduce maternal mortality, the JSY programme needs significant strengthening of the facilities through which it is implemented. Our study shows that the provision of basic and comprehensive EmOC functions in the studied districts was poor. A significant proportion (two thirds) of all births occurred in ‘less than BEmOC’ facilities. This implies that these facilities would only be able to appropriately manage relatively uncomplicated births, which might have occurred uneventfully at home. Their contribution to therefore reducing maternal morbidity and mortality is questionable, as it is precisely the women who develop complications and are most at risk who cannot be appropriately managed. While the smaller non-programme (private) sector employs a majority of qualified obstetricians and can perform caesareans, private facilities are expensive and centred in urban areas. The public sector JSY programme is, therefore, extremely important for the majority of MP’s population, and a large proportion of births occur in programme facilities. This places an increased responsibility on programme managers and the overall health system to ensure that adequate standards of EmOC are provided under the programme. Given the current situation of EmOC as reflected in this paper, it is clear that much work is needed in this direction. A demand-side programme can only be successful in the face of an adequately functional supply side; otherwise, the programme runs the risk of attracting women for whom it cannot provide a decent standard of care.

### Ethics

The study was approved by the ethics committee of R.D. Gardi Medical College, Ujjain.

### Consent to participate

Written Consent for participation in the study was taken from the respective facility and from district- and state-level health authorities.

### Consent to publish

Not applicable.

### Availability of data and materials

The authors are happy to share anonymised data related to this paper upon receiving a specific request, along with the purpose of that request. Interested parties may contact vishal.diwan@rdgmc.edu.in.

## Abbreviations

BEmOC: basic emergency obstetric care
CEmOC: comprehensive emergency obstetric care
EmOC: emergency obstetric care
JSY: Janani Suraksha Yojana
MMR: Maternal Mortality Ratio
MP: Madhya Pradesh
